# The experiences and thoughts of Turkish family physicians about COVID-19 pandemic: A qualitative study

**DOI:** 10.1080/13814788.2023.2169270

**Published:** 2023-02-14

**Authors:** Duygu Ayhan Başer, Pınar Döner Güner, Raziye Şule Gümüştakım, Ezgi Agadayi, Hilal Aksoy, İzzet Fidancı

**Affiliations:** a Department of Family Medicine, Hacettepe University School of Medicine, Ankara, Turkey; b Department of Family Medicine, Mustafa Kemal University School of Medicine, Hatay, Turkey; c Department of Family Medicine, School of Medicine, Kahramanmaraş Sütçü İmam University, Kahramanmaraş, Turkey; d Department of Medical Education, Sivas Cumhuriyet University School of Medicine, Sivas, Turkey

**Keywords:** COVID-19, family physicians’ experiences, general practice/family medicine, pandemic, qualitative designs and methods

## Abstract

**Background:**

Efforts to contain the SARS-CoV-2 virus would fall short without strong primary health care.

**Objectives:**

In this study, we aimed to determine the experiences, views and suggestions of family physicians regarding their roles, primary care health systems’ preparedness and the challenges/needs for a better organisation during the pandemic *via* in-depth exploration.

**Methods:**

Twenty-one family physicians working in different cities of Turkey participated in semi-structured interviews between 15/08/2020-21/01/2021. Convenience sampling was used. We did this qualitative study through interviews by telephone. Participants were asked seven open-ended questions. Thematic analysis was used, which included reading the transcript, identifying significant phrases and formulating meanings and validating meanings through research team discussions to reach consensus, identifying themes.

**Results:**

Ten of the participants were female and the average age of the participants was 39.5 (SD = 10.5) years. Twelve of the family physicians are specialists in family medicine. Four themes were identified: role of primary care in the pandemic, pandemic preparedness of primary care, challenges of working in primary care centres during the COVID-19 pandemics, and approaches to future pandemics.

**Conclusion:**

Our study showed that, despite unprepared primary care and undefined roles of family physicians in pandemic planning, family physicians played a significant role in pandemic management.

KEY MESSAGES

- There was not sufficient pandemic preparedness in primary care.
- There were a lot of challenges (no pandemic preparedness, indeterminate role of primary care physicians in pandemic planning, lack of guidelines on how to manage the pandemic process, etc.) at primary care pandemic management according to the opinion of family physicians.

## Introduction

The COVID-19 pandemic has caused various effects on many health systems including primary care [1]. It has been emphasised in many studies that well-planned (with universal health coverage, updated pandemic plans that include primary care, and good government and public support for the public health measures) primary care services before and during a pandemic could reduce the impacts of the pandemic [2,3].

In Turkey, many measures have been taken during the emergence of COVID-19 cases. A Scientific Board for COVID-19 was established, ‘COVID-19 Disease Guideline’ was prepared, and the Pandemic Influenza National Preparedness Plan, Pandemic Coordination Boards have been established on the national and provincial levels [4]. Health systems, including primary care services, are faced with a big stress during the COVID-19 pandemic. In Turkey, family health care centres are composed of primary care teams (a family physician with a team of one nurse and one or two medical assistants) and each team is responsible for providing preventive and therapeutic care for 1000 to 4000 people. There is no gatekeeper system in Turkish health care system, so in any condition, individuals could apply to any level of the health care system [5]. In COVID-19 pandemic primary healthcare centres are in a unique position in reaching the public in terms of suspected and confirmed COVID cases, all kinds of non-COVID health services and also other healthcare needs [6]. In order to serve optimal health services in primary care for both COVID-19 and non-COVID-19 patients during the pandemic, it is necessary to determine the pandemic preparedness of primary care, the roles of family physicians, and the challenges and needs in the primary healthcare system. Interviews with family physicians during pandemic are a way to gain better insight into the experienced enablers and barriers. However, there are few studies exploring this experience in a multidimensional manner [7–9]. Limited number of qualitative studies reported the views of family physicians [7,10–13].

The aim of the study was to determine the experiences, views and suggestions of family physicians regarding their roles, primary care health systems’ preparedness and the challenges/needs for a better organisation during the pandemic *via* in-depth exploration.

## Methods

### The research team

All researchers were family medicine academicians, and all work as family physician in primary care centres of university hospitals and treat patient. Except one (EA, female), all had experience about working as a physician during COVID-19 pandemic. All had experience in qualitative research.

### Study design

We conducted a qualitative study using a phenomenological approach to investigate the experiences, views and suggestions of family physicians during COVID-19 pandemic in Turkey.

### Setting

This qualitative study was conducted between 15/08/2020-21/01/2021 with family physicians from different cities (Ankara, Sivas, Hatay, Kahramanmaraş, Denizli, Tekirdag) in Turkey. Health facilities of physicians were situated in both rural and central areas, and offer an array of curative, preventive and promotive healthcare services.

### Sampling strategies

Study sample consisted of family physicians who actively worked in family health centres during the COVID-19 pandemic and who had at least 6 months’ experience working at the primary care level before COVID-19 pandemic. Inclusion criteria were applied to all actively worked family physicians; convenience sampling was then used to contact potential participants to inform them of their eligibility to partake. These participants had direct contact with both suspected and confirmed COVID-19 patients. These physicians were approached by the researcher (all interviews in the study were conducted by a researcher who did not know any of the study participants) *via* telephone. Their consent was asked again before starting the interviews.

### Data collection

A semi-structured interview guide, which was designed after a review of the literature, pilot interviews and discussions within the research team, was used to collect data and to serve instructions for the persons that perform the interview. We did this qualitative study through interviews by telephone. A pre-test was conducted with a family physician prior to the actual study in order to check the instrument (questionnaire) for consistency. No changes were made in the questionnaire and open-ended questions. The data collection instrument comprised two sections: The first was related to the demographic characteristics of the participants (Age, gender, years of work experience, and family medicine specialty (In Turkey all doctors who graduated from medical school can be a family physician; but if they complete their 3-year family medicine residency training, they become family medicine specialists), years of work in primary care, history of infection with COVID-19, follow up COVID-19 patient before the interview) and the second was related to experiences, views and suggestions of participants’. The questions which were used in these semi-structured interviews are presented in Box 1. With participant permission, all interviews were audio-recorded.

The mean duration of the interviews was 19 min (SD = 9.1). The aims and voluntary nature of the study were explained to family physicians, and oral informed consent was obtained before each interview. Throughout this study, we followed the Standards for Reporting Qualitative Research guidelines.

### Data analysis

We used thematic analysis. The audio recordings were transcribed verbatim. (1) Transcripts were read carefully by two researchers to get a sense of what was contained in them, (2) constant reading and re-reading of the transcript were performed to identify the underlying meaning in the interviews, (3) relevant thoughts and ideas were jotted down in each transcript, (4) this process was repeated with all the transcripts and a list of all topics were made, and (5) similar topics were clustered together to form categories and sub-categories. The interviews and original transcriptions were in Turkish. Transcripts were sent to participants for review and no further edits were required. The analysis included reading the transcript, identifying significant phrases and formulating meanings and validating meanings through research team discussions to reach consensus, identifying themes. The method described by Francis et al. was used for this study to determine data saturation [14]. In total, 23 family physicians were contacted by the researcher. We estimated 18 interviews would be needed and data saturation was tested, by conducting subsequent interviews. As new themes appeared from the new interviews, one further interview was conducted. Two family physicians refused to participate to study during data collection process. At the end of the 21 interviews (absence of any new themes emerging), data saturation was reached.

## Results

### Socio-demographic data

Twenty-one family physicians were interviewed. All participants were in direct contact with COVID-19 cases and have been working in primary care for a minimum of 6 months. The participants aged 39.5 years (SD = 10.5) (min = 25 year; max = 61 year). Ten of them (47.6%) were female and twelve of the family physicians (57.1%) were specialists in family medicine. Two of the participants (9.5%) have been infected with COVID-19.

Four themes were identified: the role of primary care in the pandemic, pandemic preparedness of primary care, challenges of working in primary care centres during COVID-19 pandemic, and approaches to future pandemics.

### Role of primary care in the pandemic

The common view of all participants was that family physicians have a very critical and important role in pandemic management. Most of the family physicians stated that the first and easiest health centre to reach during the pandemic was the family health centres, and in this respect, they have an important place in the pandemic.

‘*In terms of public health, to take a very active role in the management of the epidemic is expected from the family physicians. Family physicians, as the health personnel that the society can reach most easily, provide training to the public, preventing the spread of the epidemic, taking a role in treatment, when necessary, etc.*’ *(P15)*

*‘In other words, family physicians had a high share in the management of the pandemic and meeting the risk. Because the first-time people came to us like this: “We can’t go to hospitals, doctor, we came to you*”’. *(P18)*

In particular, they stated that family physicians have a special role in preventive medicine and counselling.

*‘I think, the primary duty of family physicians should be educating people about spreading ways of infection, ways of protection’. (P2)*

*‘…They have an important role. Education, counselling, management, monitoring… family physicians are at every stage’. (P8)*

*‘I think it may be among the duties of ensuring the continuation of primary protection services such as vaccination during the pandemic period, ensuring continuity in the management of chronic diseases, and providing referral-coordination of possible cases by following algorithms’. (P13)*

Physicians stated that job descriptions were not well defined in the pandemic:

*‘…Our job remained as a secretarial job that anyone could do. We are not effective in neither diagnosis nor treatment regulation. However, we are responsible for calling patients and preparing health reports. …We learned about current advances, treatment approaches, changes through our individual efforts’. (P1)*

*‘…However, this role could not be realized during the pandemic period. The fact that family physicians were not included in the pandemic guidelines, while developing the pandemic plan, created great difficulties’. (P4)*

Two of the physicians stated that the role of family physicians in public pandemic education is also very important and they mentioned that family physicians were pioneers in this regard.

*‘Of course, we tried to apply what we heard and our precautions as much as we could, as much as we learned at school before. …. We all started to use our masks and visors when there was no obligation for everyone to wear a mask. We warned the patients about this. We paid attention to the distance. Attention was paid to these on behalf of our family health centre*’. *(P9)*

### Pandemic preparedness of primary care

All the physicians stated that Turkey had not pandemic preparedness, especially in primary health care services.

*‘A pandemic plan should be made for primary care…’ (P4)*

*‘Family physicians were not prepared for the epidemic. There can be no individual preparation for such an epidemic, anyway, readiness should be ensured in all units through the family medicine practice. This can only be through the management. It is necessary to prepare guidelines such as how the job descriptions will be before the epidemic and how the family health centres will work…’ (P3)*

*‘We were unprepared for the pandemic as we had not encountered a serious epidemic situation before’. (P1)*

Some of the physicians stated that primary care was organised quickly afterwards.

‘*The pandemic was something we did not expect. For this reason, we can say that we were caught unprepared, but we adapted to the situation in a short time and helped our patients’. (P3)*

### Challenges of working in primary care centres during COVID-19 pandemics

All family physicians mentioned about their formal, and informal additional tasks and the impacts of these new roles on their workload and they stated that the number of patients who apply at family health centres had decreased as a result of the curfews and the fear of getting COVID-19 at beginning of the pandemic, but then number of visits were increased because patients preferred to go to the family health centres instead of hospitals. The main problems of family physicians in the pandemic were reported as loneliness of family physicians in the field, not being in an organisation, not having role definitions in pandemic guidelines, not having anyone to consult with, referral problems and a higher risk of transmission.

‘*In hospitals, the processes and algorithms are much clearer and there are many more professors and scientific resources they can access in confusion’. (P1)*

*‘The most important disadvantage of family health centres were that they were far from hospital organization, and they were alone’. (P2)*

Some physicians stated that the workload in primary care was high, but it could not be compared especially with healthcare workers who work in pandemic intensive care units.

*‘In my opinion, not keeping a service watch and having certain working hours were advantageous aspects of primary care, but there were also more disadvantages…’ (P5)*

*‘We are perhaps less tired compared to pandemic hospitals, especially I cannot compare it with intensive care’. (P8)*

*‘We certainly have had problems, but it cannot be compared with healthcare professionals who have dealt with the pandemic in hospitals. Maybe our handicap has been that we have to contact everyone without knowing who is positive and who is negative’. (P9)*

All family physicians reported that they had problems in the follow-up of patients with a diagnosis of COVID-19 in the early stages of the pandemic. The biggest problems of family physicians were the lack of a guideline developed for family physicians in the first period of pandemic, the lack of previous experience in this field, and the absence of anyone to consult patients in the family health centre.

*‘Of course, I did. We are primarily following the patient, but we are not authorized to provide a solution to the patient. The patient says that he does not have the medicine, we do not have the medicine, he is sent home because he cannot find a bed. The patient tells us about the problem, but we are not the person who can find a place’. (P8)*

*‘We do not know the disease, we do not know the symptoms, we do not know where to send it and how to examine it. It was a time of serious trouble’. (P21)*

### Approaches to the future pandemics

Some physicians stated that in a new pandemic, they will be more conscious and less anxious with their experiences in COVID-19 pandemic, while others stated that they want not to go through a pandemic process again.

*‘Now we are prepared about the epidemic management. Our infrastructure is more robust. We have written guidelines and regulations. In line with these, I hope not, but I think we will be more ready if there is a pandemic again. I think there will be no such anxiety and the public will be more conscious’. (P7)*

*‘Of course, although there will be changes in the measures to be taken depending on what the factor is, I will approach it more experienced and calmer. I would be more demanding and oppressive legally in order to ensure that the functioning of family health centres is more effective and aimed at protecting health personnel in the long run’. (P20)*

*‘I hope that if there is a pandemic again, I will not be a family doctor in those days’. (P3)*

*‘The experience we gain allows me to take action faster. I am more careful in protecting myself and family health centre staff. I make plans and arrangements so that the management of non-pandemic diseases is not disrupted’. (P7)*

One participant stated that she was afraid of experiencing the burnout earlier in future pandemics that she experienced during the COVID-19 pandemic. Other participant said that he would be more careful in protecting herself and other healthcare personnel.

*‘There will be no change in patient management in a new pandemic. But I am afraid of experiencing the health burnout we experienced earlier during the first pandemic’. (P6)*

## Discussion

### Main findings

The results of this qualitative study showed that although the indeterminate role of family physicians in pandemic planning, they played a significant role in various conditions based on their opinions. There was not sufficient pandemic preparedness in primary care and there were a lot of challenges in primary care pandemic management according to the opinion of family physicians. As a result of this, family physicians found themselves in a position of developing their own solutions during the pandemic in Turkey. With this pandemic, the development of guidelines and prevention algorithms for primary care is stated as a good process for future pandemics.

### Role of primary care in the pandemic

WHO defined the role of primary care in the pandemic as follows: ‘*Primary care plays a significant role in gatekeeping and clinical responses: differentiating patients with respiratory symptoms from those with COVID-19, making an early diagnosis, helping vulnerable people cope with their anxiety about the virus, and reducing the demand for hospital services*’ [15]. In our study, the common view of all family physicians was that family physicians have a very critical and important role in pandemic management, and they have a special role in preventive medicine and counselling. However, the indeterminate role in pandemic planning was the biggest problem of them. In studies from different countries, there were different responses [13,16]. In a study from Ares-Blanco et al. they described the role of primary care in 12 European countries in relation to the COVID-19 pandemic and stated that all countries provided COVID-19 information through telephone lines and websites to their citizens and this application facilitated the pandemic preparation [16].

### Pandemic preparedness of primary care

The importance of pandemic preparedness has been highlighted in recent years [17]. Preparedness plans consist of public health capacity building and taking measures about surveillance, communication, vaccination services, and maintenance of an inventory of antiviral drugs [18]. Many countries were caught unprepared by the COVID-19 pandemic [19]. In an article from Huston and colleagues, who provide a commentary on the primary care response to COVID-19 in six well-resourced countries (Australia, New Zealand (NZ), Canada, the Netherlands, the United Kingdom (UK) and the United States (US)), they stated that countries differed in terms of pre-existing universal health coverage, pandemic readiness, and the level of government and public support for public health measures. Of the six countries, Australia and NZ were stated as the best prepared, because of the universal health coverage, up-to-date pandemic plans and identified primary care. The US was stated as worst, it does not have universal health coverage and despite the updated pandemic plan (2017), primary care had no defined role [2]. In our study, physicians stated that there was no pandemic preparedness both at the primary care level and at all other health services in our country. This has been identified as an important problem that affects pandemic management and causes confusion. One of the first things to be done in order to bring the pandemic management to the best level in primary care is to consider the primary care as a whole and to address the problems of the family physicians who see active patients in the field, as well as the managers and educators. In our study, some of the physicians stated that primary care was organised quickly afterwards. Especially, the increased number of admissions to primary care could provide quick organisation of primary care.

### Challenges of working in primary care centres during COVID-19 pandemics

In our study, all physicians mentioned additional tasks and the impacts of these new roles on their workload. Our results about the challenges of working in primary care were consistent with previous studies. When evaluated in terms of COVID-19 pandemic, extreme workload, rapidly changing practice environment, lack of personnel protective equipment, limited information, insufficient training, role ambiguity of physicians, and insufficient cooperation with other healthcare workers were specified as challenges of primary care in different countries [20–23]. Loneliness of family physicians in the field, not being in an organisation, and not having role definitions in pandemic guidelines were the other mentioned problems of family physicians, specifically reported in our study. In Turkey, family physicians faced serious difficulties especially at the beginning of the pandemic both due to the lack of guidelines on how to manage the pandemic process and the lack of personal protective equipment. There was an ambiguity in the roles of family physicians at the beginning of the pandemic in our country and family physicians did not know what to do at the onset of the pandemic, how they should protect themselves and their patients, and how they should manage the pandemic. Hospitals were initial places of interest for corona fight and all hospitals were accepted as pandemic hospitals including private ones. Because of this, patients other than COVID 19 have started to prefer family health centres much more for all kinds of complaints, because they have difficulty accessing health centres outside the family health centre and because of the fear of COVID 19 transmission. In other words, family physicians continued to provide services to patients from all ages, with all complaints or diseases without any restrictions during COVID-19 pandemic. In accordance with our study results, the study carried out by Yasin et al. in Turkey revealed that even though primary care physicians were burdened with unprecedented increased workload during the COVID-19 pandemic and the uninformative management of the COVID-19 pandemic [23]. Although the accessibility of primary care was a good opportunity for patients during pandemic, this situation has caused problems for the primary healthcare professionals due to lack of planning. Besides, family physicians monitor the registered patients who meet the COVID 19 case definition and who come into contact with these cases by telephone on a daily basis [24,25]. Although the role of family physicians in pandemic is very important, the lack of planning and organisation in this process caused difficulties for family physicians except for the increased workload as found in our study. In our country, the fact that patient referrals were never made systematically, and no information was provided on this subject may have caused these problems.

In line with other studies in primary care, family physicians reported difficulties on limited access to practical information, guidelines on how to manage patients with COVID-19 [13,26]. As can be understood from the participants’ comments on our study; the guidelines, algorithms and planning prepared during the COVID-19 pandemic process will help to be more ready for a pandemic that may develop in the near future. However, it is necessary to clarify the primary care pandemic plans and to form pandemic preparedness guidelines for primary care by the ministry and non-governmental organisations.

### Approaches to the future pandemics

There were various answers for approaches to future pandemics. Some of them stated that COVID-19 pandemic was an important experience in this regard. In a study from the United Kingdom, authors stated that previous experiences and a strong health service of family doctors provided rapid adaptation to new COVID-19 pandemic [27]. In out county, there was no experience of primary care related with pandemic, so with this pandemic, the development of guidelines and the preparation of prevention algorithms in primary care conditions have been an important process for future pandemics. In particular, the fear of going into burnout earlier in future pandemics was an important and remarkable finding of this study. In a study of Baptista et al. they stated that physician burnout in primary care was high and had increased during the pandemic [28].

### Limitations

This study determined thematic saturation through the widely accepted empirical approach. Despite its popularity, there are a few limitations. Thematic saturation was not quantitatively assessed due to the challenges of measuring the degree of saturation statistically; it is dependent on the researcher’s subjective judgement to determine when thematic saturation has been achieved. (In this study, the sample cover different profiles of physicians according to age, and working time as a family physician. However, the main criteria for inclusion was working as a family physician during the pandemic. So these differences may have influenced the comparison with past experience. The sampling technique was very beneficial, however purposive technique should be more appropriate. Regional distribution of participants could be noticed.

## Conclusion

Our study showed that, despite unprepared primary care and undefined roles of family physicians in pandemic planning, family physicians played a significant role in pandemic management. The importance of better structuring of primary care has been emphasised due to reasons such as being the first place of application and increasing workload. As a conclusion, updated pandemic plans with defined roles of primary care and good primary care organisation are the most important steps in pandemic management. Qualitative and quantitative studies to be conducted with a larger sample can reveal more clearly what needs to be done in preparation for the future pandemic.
